# Fertility costs of cryptic viral infections in a model social insect

**DOI:** 10.1038/s41598-022-20330-4

**Published:** 2022-09-23

**Authors:** Abigail Chapman, Esmaeil Amiri, Bin Han, Erin McDermott, Olav Rueppell, David R. Tarpy, Leonard J. Foster, Alison McAfee

**Affiliations:** 1 Department of Biochemistry and Molecular Biology, Michael Smith Laboratories, University of British Columbia, Vancouver, BC Canada; 2 Delta Research and Extension Centre, Mississippi State University, Stoneville, MS USA; 3 Institute of Apicultural Research, Chinese Academy of Agricultural Sciences, Beijing, China; 4 Department of Entomology and Plant Pathology, North Carolina State University, Raleigh, NC USA; 5 Department of Biological Sciences, University of Alberta, Edmonton, AB Canada

**Keywords:** Proteomics, Entomology

## Abstract

Declining insect populations emphasize the importance of understanding the drivers underlying reductions in insect fitness. Here, we investigated viruses as a threat to social insect reproduction, using honey bees as a model species. We report that in two independent surveys (N = 93 and N = 54, respectively) of honey bee (*Apis mellifera*) queens taken from a total of ten beekeeping operations across British Columbia, high levels of natural viral infection are associated with decreased ovary mass. Failed (poor quality) queens displayed higher levels of viral infection, reduced sperm viability, smaller ovaries, and altered ovary protein composition compared to healthy queens. We experimentally infected queens with Israeli acute paralysis virus (IAPV) and found that the ovary masses of IAPV-injected queens were significantly smaller than control queens, demonstrating a causal relationship between viral infection and ovary size. Queens injected with IAPV also had significantly lower expression of vitellogenin, the main source of nutrition deposited into developing oocytes, and higher levels of heat-shock proteins, which are part of the honey bee’s antiviral response. This work together shows that viral infections occurring naturally in the field are compromising queen reproductive success.

## Introduction

Amidst a backdrop of widespread insect declines^1–6^ and fluctuating populations^7,8^, it is vitally important to better understand the impacts of interacting biotic and abiotic stressors on insect physiology^9^. Pesticide exposure and climate change are often cited as drivers of insect decline^9–13^, but biotic drivers, such as viruses, are comparatively understudied, despite some viruses exhibiting broad host ranges^14^ with the potential for widespread impacts across species. Stressors that reduce fertility are particularly worrisome, especially for social insects, as damage to a single individual (the queen) can weaken the fitness of the entire colony. Additionally, extreme temperatures can have immunomodulatory effects in some insects^15,16^; therefore, the stressors of climate change and viral infections may actually interact to have synergistic effects, further underscoring the need to better understand the effects of both stressors^17^.

Viruses that affect managed species like honey bees (*Apis mellifera*) can infect a broad range of insect host species, creating the potential for pathogen spillover to native species pollinating in the same regions^18–25^. Infection with Israeli acute paralysis virus (IAPV) and Kashmir bee virus (KBV), which infect both *Apis* and non-*Apis* pollinators, results in slower colony start-up and a reduction in egg laying in *Bombus terrestris*^26^. Furthermore, high titres of deformed-wing virus (DWV) are associated with extreme cases of ovarian degeneration in honey bee queens^27^, but there is a need for more research in this area.

Beyond the symptomatic impacts on insect health, virus infections also have the potential to indirectly reduce fecundity, even in the absence of overt symptoms, through a trade-off between reproductive ability and immune activation^28^. Reproduction and immune processes, whether induced by a pathogen or constitutively maintained, are both energetically demanding. Given a finite resource supply, an individual may not be able to sustain both fully at the same time^28^. Across multiple insect orders, mated female insects tend to have reduced immunity compared to their unmated counterparts^28^. Correspondingly, immune-challenged individuals frequently show a reduction in reproductive output measured as reduced overall fecundity^29^, reduced protein in both ovaries and eggs^30,31^, reduced oviposition rate^31,32^, and reduced viability of stored sperm^33,34^. We have previously shown that lysozyme, an immune effector, is negatively correlated with stored sperm viability in honey bee queens and that failing queens, even without symptoms of viral infection, have significantly lower sperm viability and higher titers of black queen cell virus (BQCV) and sacbrood virus (SBV)^35^. This suggests that reproduction and immune activation, as a result of pathogenic challenge, are negatively associated in honey bee queens, at least in terms of sperm maintenance. Furthermore, a potential immune-reproduction compromise is particularly relevant for a honey bee queen, whose physiology is tailored for laying eggs and little else; their ovaries make up about one third of their body mass, and they can lay > 1000 eggs a day, which is roughly equivalent to their own body weight^36,37^. This is a massive investment of resources in a singular reproductive process, contrary to sperm maintenance, which is achieved mainly by enzymatic suppression of reactive oxygen species in the spermatheca and creating the conditions that sustain sperm in a metabolically quiescent state^38,39^. Indeed, if reproduction-immunity trade-offs apply to sperm storage, as has been suggested in honey bees and other insects^28,33–35,40^, they would most likely be due to collateral damage of immune effectors, such as reactive oxygen species, rather than resource-allocation. Additionally, a queen’s resource investment in ovary size is highly plastic and responds dramatically to external stressors like nutrient availability^37^. Thus, viral infections could negatively impact fertility either through direct effects of infecting reproductive tissue or compromised allocation of resources associated with systemic infections.

Here, we investigated the relationship between viral infection and reproductive physiology in honey bees, a model social insect. We measured the ovary masses of queens rated as ‘failed’ and ‘healthy’ by beekeepers which had natural variations in viral infection. In two independent populations, we identified and validated a negative relationship between viral infection and ovary size. We measured ovarian protein investment associated with viral infection using quantitative proteomics and confirmed the upregulation of antiviral proteins in virus infected queens. Finally, we have confirmed the causal, rather than correlative, effect of virus infection on ovaries by experimentally infecting queens with IAPV. This work suggests that pathogenic infections negatively impact reproductive quality and fitness in a model insect, supporting the idea that infection may be a significant driver of declining insect health.

## Results and discussion

### Failed queens have smaller ovaries and reduced sperm viability

**Figure 1:**
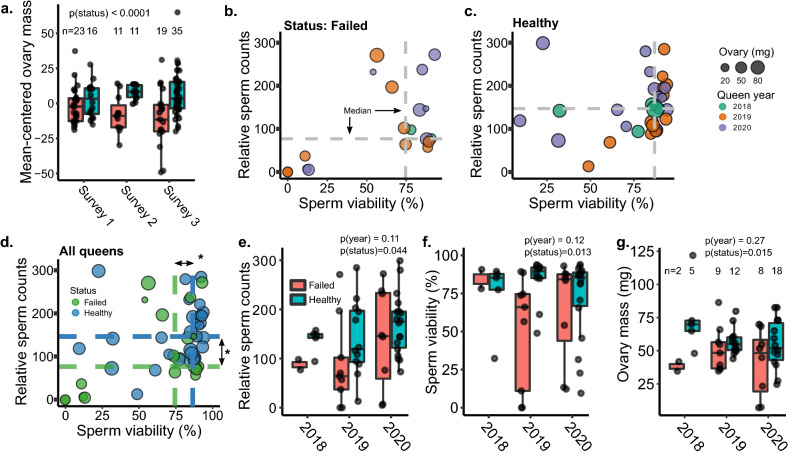
Fertility metrics of failed and healthy queens. In all cases, years indicate the year the queen was reared. (**a**) Ovary masses of failed and healthy queens collected across three different surveys conducted in British Columbia and Pennsylvania (analyzed using a linear mixed model with status as a fixed effect and source location as a random effect). Because the average ovary size differed between surveys, data were mean-centered by survey prior to analysis to better highlight the effect of status. Boxes represent the interquartile range, bars indicate the median, and whiskers span 1.5 times the interquartile range. (**b**) Queens rated as ‘failed’ (spotty brood pattern, drone layer, or dwindling adult population) or (**c**) ‘healthy’ (contiguous worker brood patterns, medium-strong adult population) by local beekeepers in British Columbia were collected in the summer of 2020. Sperm viability and sperm counts were determined by fluorescent imaging, and wet ovary weight was measured on an analytical balance. (**d**) Statistical analyses on data presented in (**b**) and (**c**) were conducted using either a linear mixed model (ovary mass and sperm counts) or a generalized linear mixed model fitted by maximum likelihood (sperm viability; see Table 2 for details). In the statistical models, queen age (0, 1, or 2 years, which corresponds to queens reared in 2020, 2019, and 2018, respectively) and health status (healthy or failed) were included as fixed effects and source location was included as a random effect. Asterisks indicate statistical significance (*p* < 0.05), with exact p values given in panels (**e**–**g**). (**e**–**g**) Same data as in (**d**) but separated by the year in which the queen was reared (i.e., a 2018 queen was 2 years old).

In order to investigate if small ovary size is a general feature of queens with poor reproductive output (described by beekeepers as “failing”), we compared the ovary masses of failing and healthy queens collected from beekeeping operations in three independent queen surveys (denoted as field surveys 1–3) conducted in multiple geographical locations (described in Table 1). We found a highly significant reduction in ovary mass (linear mixed model; t =  − 4.37, df = 104, *p* < 0.0001) in failed queens across surveys (Supplementary Table S1 and Fig. 1a), indicating that small ovaries are a widespread phenotype associated with poor fecundity, as described by beekeepers. Whether this is a causal factor or merely a symptom remains unclear, but underscores the relevance of ovary mass as an important queen quality metric.

**Table 1:** Summary of queen surveys.

Survey	Year collected	Location	Queens age-matched?	Quality metrics acquired
Survey 1	2019	Throughout British Columbia, subset including only queens from south-central BC^a^	No	Ovary mass, viral abundance, sperm viability, absolute sperm counts
Survey 2	2018	Pennsylvania	Yes	Ovary mass
Survey 3	2020	Greater Vancouver, BC	Yes	Ovary mass, viral abundance, sperm viability, relative sperm counts

^a^These queens were chosen from a previously published dataset^35,41^ because failed and healthy queens were included in the same shipment, eliminating potential extraneous variables associated with shipping.

In our previous queen survey (Survey 1), we also found that queen failure was associated with reduced stored sperm viability and sperm counts (not recorded for Survey 2)^35^. However, the ages of failed queens were unknown, leading to questions over whether differences in queen quality metrics were linked to queen failure or old age. We therefore collected an additional n = 54 queens (19 failed and 35 healthy), comprising Survey 3, with known ages, the oldest of which were 2 years old, all from our local region in British Columbia to avoid extraneous effects of shipping. We found that sperm viability (generalized linear mixed model, family = binomial; z =  − 2.65, *p* = 0.008) and relative sperm counts (linear mixed model; t =  − 2.32, df = 44, *p* = 0.025) were again significantly lower in failed queens, even when accounting for age as a fixed factor and queen source as a random effect (Fig. 1b–f), corroborating our previous studies^35,42^. See Table 2 for complete statistical reporting and Supplementary Table S2 for the underlying data. In this survey, failed queens also had significantly smaller ovaries (linear mixed model, t =  − 2.33, df = 43, *p* < 0.023) (Fig. 1g).

**Table 2:** Statistical parameters.

Data set	Response	Model	Fixed effect	Estimate	df	|t/z/F|	*p*
Ovary masses of queens in field surveys 1–3^a,b^	Ovary mass	Linear mixed model (lme)	Health status	− 12.36	104	− 4.37	< 0.0001***
Field survey 1 queens (n = 93)^a^	Ovary mass	Linear mixed model (lme)	DWV-A	− 1.61	88	− 2.15	0.035*
			BQCV	− 1.28		− 1.70	0.093
			SBV	− 0.32		− 0.34	0.73
			Health status	− 5.22		− 1.51	0.13
		Linear mixed model (lme)	Total viral load	− 2.26	90	− 3.53	< 0.001***
			Health status	− 3.30		− 1.22	0.23
Field survey 3 queens (n = 54)^a,c^	Ovary mass	Linear mixed model (lme)	DWV-B	− 0.61	49	− 0.35	0.73
			DWV-A	− 5.17	49	− 1.81	0.077
			LSV	2.62	48	0.49	0.63
			Health status	− 12.43	49	− 2.33	0.024*
		Linear mixed model (lme)	Total viral load	− 2.07	49	− 1.37	0.18
			Health status	− 12.65	43	− 2.34	0.023*
	Sperm viability	Generalized linear mixed model (glmer)	Age	0.04	n/a	2.65	0.06
			Health status	− 0.066		− 1.78	0.008**
			Total viral load	0.0074		1.15	0.25
	Sperm counts	Linear mixed model (lme)	Age	− 17.87	44	− 1.21	0.23
			Health status	− 49.07		− 2.32	0.02*
			Total viral load	9.28		1.49	0.14

^a^Models included queen source location as a random effect.

^b^Survey 2 contributed only ovary mass data, and not sperm metrics. Survey 2 data were analyzed in conjunction with ovary mass data from surveys 1 and 3.

^c^Generalized linear mixed models, used for the sperm viability model for survey 3, do not report degrees of freedom. For this model there were 54 observations and 7 groups.

### Ovary size is inversely linked to viral abundance in naturally infected queens

Queen honey bees are readily infected by many pathogenic agents^27,43–45^, and most commonly infected with sacbrood virus (SBV), black queen cell virus (BQCV), and deformed-wing virus (DWV)^35,43^. We previously found that the failed queens from Survey 1 have higher copy numbers of SBV and BQCV RNA^35^.We therefore hypothesized that ovary size is linked to viral load, with the rationale that viral infection could either directly impact ovary function by infecting the tissue, or indirectly impact ovaries by shunting resources into immune activation while depleting resources available to invest in ovarioles.

Using the previously published viral analysis of queens from Survey 1 (n = 93)^35^, which was acquired by absolute quantification using qPCR and reported as copies per ng of total RNA, we tested if viral load was a predictor of ovary mass. Viral abundance was not significantly associated with the other fertility metrics of sperm viability and sperm counts that we collected (Table 2). A subset of these queens was previously analyzed for 7 of the most common honey bee viruses but only DWV-A (DWV-B was not specifically included), SBV, and BQCV were detected, so the remaining queens were analyzed for just these three. We used two models for analysis: the first with the copies of each individual virus as fixed effects, and the second using a “total viral load” in which the counts of each virus were summed together. We were interested in the effects of an additive total viral load because we hypothesized that the changes in ovary mass are caused by viral infection generally, and not driven by the effects of one particular virus. For both, we used a linear mixed effect model (lme package in R) with queen source (breeder) as a random effect, and queen health status (failing or healthy) and virus counts (separately or added) as fixed effects. See Table 2 for complete statistical reporting and Supplementary Table S3 for the underlying data.

**Figure 2:**
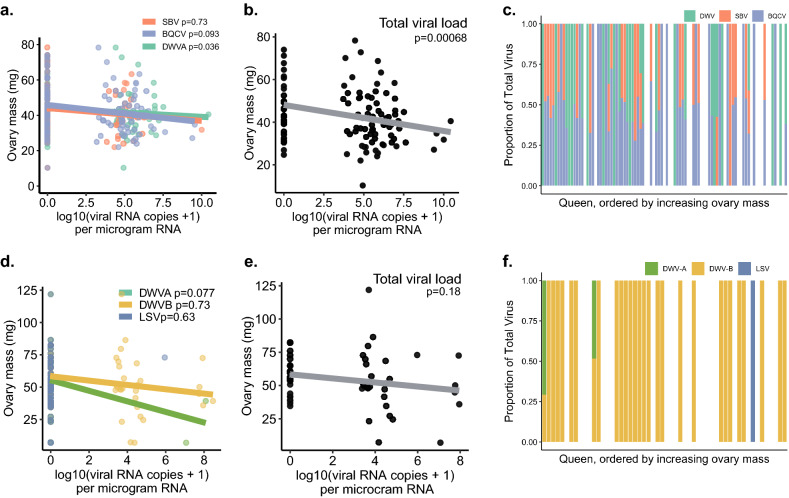
Relationships between viral RNA copies and ovary mass. See Table 2 for complete statistical details. (**a**) Survey 1 (previously published; see Table 1 for details) included viral RNA copies for deformed-wing virus A (DWV-A), sacbrood virus (SBV), and black queen cell virus (BQCV), using head tissue samples. (**b**) Viral RNA copies for each virus were added to produce the variable “Total viral load” and analyzed as a fixed effect instead of individual virus copies. (**c**) Proportion of each virus in each queen, ordered by increasing ovary mass. (**d**-**f**) The same relationships from a validation data set (Survey 3) of n = 54 queens analyzed for 8 different viruses in the thorax using a different laboratory service. Queens were mainly infected with DWV-B (non-detected viruses not shown), with sporadic DWV-A and Lake Sinai virus (LSV). For (**a**) and (**d**) data were analyzed using a linear mixed effect model with queen source as a random effect, and the copies of each virus and health status (healthy vs. failing) as fixed effects. Regression lines shown only for viruses with > 1 non-zero point. (**e**) The summed total viral load was analyzed as a fixed effect instead of the individual virus copies.

When the counts of each of the three viruses were used as predictors individually (Fig. 2a), we found that only DWV was statistically significant (estimate =  − 1.6111, df = 88, t =  − 2.146, *p* = 0.03), although BQCV had a similar effect size (estimate =  − 1.2779, df = 88, t =  − 1.698, *p* = 0.09). In the combined model (Fig. 2b) total viral load was significant with a greater effect (estimate =  − 2.2598, df = 90.0000, t =  − 3.530, *p* = 0.0007). Figure 2c illustrates the relative proportion of each virus in each queen, which reveals no obviously discernable pattern between each virus and ovary size. Additionally, when comparing the two models using Akaike Information Criterion scores, the total load model is a better fit, indicating that combined viral load is a better predictor of ovary mass than the counts of any one virus. Combined, these illustrate that increased viral load is significantly associated decreased ovary mass, and that this is not obviously due to the effects of one specific virus, but more likely due to viral infection in general.

We then investigated this relationship with the n = 54 queens from Survey 3, which we sampled independently from different apiaries, and whose viral abundances were analyzed by a different laboratory and reported as copies per ng total RNA (data in Supplementary Table S4; Fig. 2d,e). Despite a much smaller sample size, identifying an overall lower range of viral abundance, and not identifying two of the three most common viruses present in queens (BQCV and SBV) (which may be due to different laboratory methods), we observed the same trends as for Survey 1. Though none of these relationships were significant, observing the same correlation in an independent set of samples, with different viruses quantified from a different tissue, provides further evidence that this is a biologically relevant phenomenon. Future studies with larger sample sizes and uniform viral quantitation methods could serve to better illuminate this relationship between natural infections.

**Figure 3:**
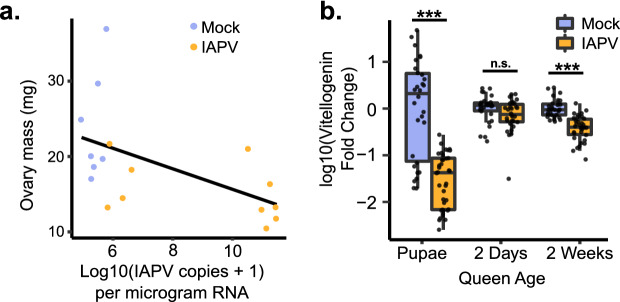
Ovary mass and vitellogenin transcription are significantly decreased after IAPV injection. (**a**) Mated, age-matched queens were injected with IAPV or a mock (buffer only; n = 10 each). After 65 h the ovary mass of infected queens was significantly reduced. Data was modeled using a simple linear model, *p* = 0.024. (**b**) The transcript for vitellogenin is downregulated in the abdomens of queens experimentally infected with IAPV at two days before emergence and two weeks post-emergence (after CO_2_ ovary activation). The fold-change in gene expression was calculated using the 2^−∆∆Ct^ method. Pairwise comparisons of gene expression were evaluated using the Wilcoxon Rank Sum test (n.s. = not significant, ***p < 0.0001).

To confirm that viral infection is causally associated with the reduced ovary size of failed queens and not merely a result of failed queens potentially being more susceptible to infection, we injected age-matched, mated queens with IAPV. We chose IAPV because it naturally infects honey bee queens, but has a low frequency of infection^43^, so experimental infection is unlikely to confound with natural abundance. After just 65 h, we found that queens with higher levels of IAPV had significantly smaller ovaries (Fig. 3a) (simple linear model: estimate =  − 1.36, t =  − 2.51, df = 15, *p* = 0.024), echoing our results from the field. There appeared to be contamination of the mock-injected queens with IAPV so we have analyzed the data as a continuous relationship, rather than as a comparison between groups for transparency. The number of IAPV copies was confirmed by absolute quantification using qPCR, and the mock-injection queens were additionally checked for DWV, SBV, and BQCV, of which there was no detectable infection in any of the queens.

Two caveats to this experiment should be noted, though. The first is that infection by injection has previously been shown to increase mortality, viral titre, and expression of immune effectors relative to the oral infection route in worker honey bees with BQCV^46^. This means that the responses we observed here are likely to represent a more extreme response than from other the other routes of transmission (oral, vertical, or sexual), though at this time, evidence for which of these represents the most common “natural” route of infection for adult queens is lacking. Secondly, assessing the mock-injected queens for DWV, SBV, and BQCV allowed us to broadly conclude that the levels of these viruses in this population of queens is low. However, we cannot rule out the possibility that these (or other) viruses may have been present in one or more queens in the IAPV-injection group, which could potentially lead to interactive effects between viruses^47^.

In a separate experiment, we investigated vitellogenin gene expression in the abdomens of IAPV-injected queens relative to mock-injected queens at three different ages: two days before emergence (pupae), two days after emergence, and two weeks after emergence (with CO_2_ treatment to stimulate ovary activation and egg laying^48^). The success of the infections was confirmed by measuring IAPV copies using qPCR (Supplementary File 1). In pupal queens and queens two weeks after emergence (after CO_2_ ovarian stimulation), we found that expression of vitellogenin was significantly reduced by IAPV infection (Fig. 3b). Vitellogenin, the major egg yolk protein precursor, is one of the most significant sources of nutrition for developing oocytes, where it is deposited in massive quantities. Much of the mass of a queen’s ovary is made up of developing eggs, therefore a decrease in vitellogenin expression is consistent with a reduction in ovary mass and together represents a reduction in the resources being invested into producing or provisioning eggs. Vitellogenin is also responsible for the binding and transport of immune elicitors (i.e., bacterial cell wall components) during transgenerational immune priming^49,50^. So, this reduction in vitellogenin could have knock-on effects for the success of the queen’s progeny by reducing her capacity to provide them with immune priming.

### Ovaries of failed queens have altered protein composition

**Figure 4:**
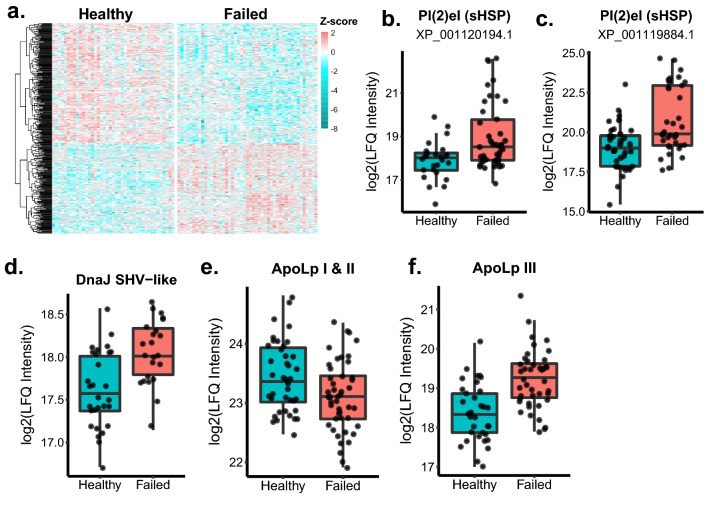
Changes in ovary protein expression and vitellogenin. Protein abundance was measured via LC–MS/MS using label-free quantitation (LFQ). Analysis was done on proteins identified in at least 10 samples and expression is reported as log2-transformed LFQ intensity. We used limma with status (failed vs. healthy), ovary mass, and total viral counts as fixed effects for n = 88 queens (41 healthy and 47 failed) to identify the differentially expressed proteins shown in (**a**), (**b**), and (**c**). Exact sample sizes may differ due to missing values for some proteins. The false-discovery rate (FDR) was controlled using the Benjamini–Hochberg method (5% FDR). (**a**) Protein expression patterns in ovaries of healthy and failed queens. Only proteins differentially expressed and quantified in 75% of samples are shown (387 proteins). (**b**) Proteins associated with the antiviral response are upregulated in failed queens (sHSP (XP_001119884.1): t = 3.8, adjusted *p* = 0.003; sHSP (XP_001120194.1): t = 3.3, adjusted *p* = 0.01; DnaJ: t = 4.9, adjusted *p* = 0.0003). (**c**) Apolipophorins I/II are involved in lipid transport and are downregulated in failed queens (t =  − 2.59, adjusted *p* = 0.048). Apolipophorin III is an important protein for immune function and lipid transport and is upregulated in failed queens (t = 5.25, adjusted *p* < 0.0001).

To determine if the changes we observed in the ovaries of failing queens were associated with altered patterns of protein expression consistent with a disruption in egg production, we performed quantitative proteomics on the ovaries of n = 88 queens collected in British Columbia from Survey 1. We found that 415 proteins, around 20% of the total number of quantified proteins, were differentially expressed between failing and healthy queens at a false discovery rate of 5% (Benjamini Hochberg method; Fig. 4a). Many of the proteins that were downregulated in failing queens are associated with metabolic processes, but no gene ontology (GO) terms were significantly enriched among the differentially expressed proteins. This could be due to the poor GO characterization of many honey bee proteins, as we have previously described^35^. However, among these differentially expressed proteins are several indicators of an increase in immune activity. One of the top proteins decreased in failed queens was prohibitin (XP_624330.3), a highly multifunctional protein associated with cellular proliferation^51^, the regulation of follicular development in mammalian ovaries^52^, and a mediator of viral entry into insect cells^53^. Furthermore, heat-shock proteins have antiviral activity in insects^54,55^, and our data are consistent with that role in queens. We found that two small heat shock proteins (protein lethal(2) essential for life), XP_001119884.1 and XP_001120194.1, as well as dnaJ homolog shv (XP_006569897.2) were upregulated in failed queens (Fig. 4b–d). Despite sharing the same name (protein lethal(2) essential for life), these proteins do not share tryptic peptides > 6 residues long; therefore, their quantification was not influenced by shared peptide sequences. DnaJ homolog shv was also previously reported to be associated with the antiviral heat-shock response in workers^55^.

Studies in Lepidopterans and Orthopterans have shown that the protein apolipophorin III (apoLp-III) is involved in both the immune system and metabolism^56^. When in its monomeric form, it acts as an immune surveillance pattern-recognition receptor with the ability to activate the immune response. We previously found that apoLp-III is significantly co-expressed with a cluster of predominantly innate immune proteins in the spermathecal fluid^35^, lending confidence to its involvement in immune processes in queens. But, when combined with high-density apolipophorin (apoLp-I/II), it acts as a lipid transporter, shuttling lipids liberated from the fat body and from the digestive system around the body^57^. One of the most significant destinations of these lipids in reproductively active female insects are the developing oocytes, where they will make up 30–40% of each egg’s dry weight^58^. These lipophorins could be a molecular switch governing both the movement of resources and immune function, and we predicted that if increased viral loads are a feature of failed queens, then they should have increased levels of apoLp-III to promote immune function. Additionally, we predicted that apoLp-I/II, which is constitutively involved in lipid transport and juvenile hormone signalling, would be reduced in the smaller ovaries of failing queens which are likely producing fewer eggs. We found that apoLp-III was indeed upregulated in failed queens, and apoLp-I/II was downregulated (Fig. 4e,f), suggesting that lipid transport to developing eggs is reduced in failing queens.

### Heat-shock proteins are upregulated in virus-infected queens

To gauge whether the levels of viral infection in the field were sufficient for the queens to launch an antiviral response, we investigated expression of putative antiviral proteins. Virus-infected workers express elevated levels of antimicrobial peptides (*e.g., defensin, hymenoptaecin*); however, these peptides’ molecular properties are not favourable for quantitation by shot-gun proteomics without specialized enrichment strategies. Additionally, we have not been able to identify or quantify several of the other proteins classically involved in the antiviral response, including Dicer and Argonaute-2, in these or similar samples. Previous work has demonstrated that in fruit flies and worker honey bees, heat-shock proteins, which are more easily quantified by proteomics, are an important part of the antiviral defense^54,55,59^. Virus-infected workers also upregulate heat-shock protein mRNA (*pl(2)el*, *hsp70-3, hsp70-4*, *hsp83-like*, and *hsp90*), and workers whose heat-shock proteins were experimentally induced by temperature stress shortly after infection had 74–90% lower viral titers compared to non-induced controls^55^. We therefore hypothesize that antiviral heat-shock protein expression is also correlated with viral load in queens.

**Figure 5:**
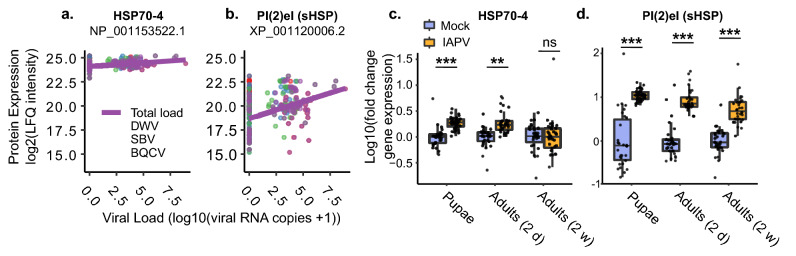
Heat-shock proteins associated with natural and experimental viral infections. (**a**, **b**) We analyzed previously published proteomics data using limma, including health status, sperm counts, and log transformed total viral loads as fixed effects (n = 88 queens had complete proteomics with no missing values in continuous covariates). Individual virus types are shown, but only total titers were statistically analyzed. *p* values were corrected for multiple hypothesis testing by Benjamini–Hochberg correction (see Table 3 for statistical summaries of these and other significant proteins). (**c**, **d**) The transcripts of two heat-shock proteins found in the spermathecal fluid of naturally infected queens and previously identified in the antiviral response in workers are upregulated in the abdomens of queens experimentally infected with IAPV. The fold-change of gene expression was calculated using the 2^−∆∆Ct^ method. Pairwise comparisons of gene expression were evaluated using the Wilcoxon Rank Sum test. ns: not significant; **p < 10^−7^; ***p < 10^−10^.

We first analyzed previously published quantitative proteomics data obtained from spermathecal fluid samples of the queens from Survey 1 with varying degrees of viral infection^35,41^. Honey bee viruses can be transmitted sexually, so spermathecal fluid is a reasonable tissue in which to look for an antiviral response^60^. Our statistical model included sperm counts, health status, and log-transformed total viral RNA copies as fixed effects. We found that six proteins were non-significantly correlated with log-transformed total viral RNA copy numbers (adjusted p-values between 0.054 and 0.082) (Table 3), half of which are heat-shock proteins: heat-shock protein 70 cognate 4 (HSP70-4) and two other small heat-shock proteins (sHSPs) XP_001120006.2 and XP_001119884.1. As we predicted, all three HSPs positively correlate with viral abundance (HSP70-4 and sHSP XP_001120006.2, which have been previously associated with the worker bee antiviral response^55^, are depicted in Fig. 5a,b). The other three proteins—a transmembrane protease, zinc carboxypeptidase, and arylsulfatase-B—have not been previously linked to viral infection in honey bees. Notably, the two sHSPs were previously proposed as candidate diagnostic markers for heat-stress and were significantly upregulated in failed queens relative to healthy queens^41^; here, we show that they are linked to viral abundance even when health status is included as a fixed effect. This observation unfortunately negates their utility as heat stress biomarkers due to confounding virus infection.

**Table 3:** Statistical summaries of proteins correlating with total viral loads.

Protein	Common name	|t|	*p*	Adjusted p*
XP_016771183.1	Transmembrane protease serine 11B like	4.48	0.0000355	0.054
XP_001120006.2	Protein lethal(2) essential for life (small HSP)	4.17	0.0000778	0.054
NP_001153522.1	Heat shock protein 70 cognate 4	4.04	0.000114	0.054
XP_623922.2	Zinc carboxypeptidase	4.05	0.000136	0.054
XP_006569022.1	Arylsulfatase-B	4.81	0.000101	0.054
XP_001119884.1	Protein lethal(2) essential for life (small HSP)	3.82	0.000246	0.082

*Benjamini Hochberg method.

To validate that these HSPs are part of the antiviral immune defense in queens we performed experimental infections with IAPV. We selected the two heat shock proteins that we identified as upregulated in naturally virus infected queens that overlapped with the previously reported list of HSPs associated with the antiviral response in workers: protein lethal(2) essential for life (XP_001120006.2, Pl(2)el) and heat-shock protein 70 cognate 4 (Hsp70-4)^55^ (all primer sequences are available in Supplementary Table S5). Pl(2)el and HSP70-4 were significantly upregulated after viral infection in queens of all three ages, with the exception of two-week-old queens, in which HSP70-4 was not differentially expressed (Fig. 5c,d, Supplementary Table S6). The fact that HSP70-4 was not upregulated in queens whose ovaries had been stimulated to develop may be because insects which have mated or begun to reproduce often demonstrate a reduced ability to mount an immune response relative to their unmated counterparts^28^, and trade-offs involving the immune response often involve a complicated reconfiguration in which different components of the response might become up or downregulated^61^.

## Conclusion

These data show that viral infection is impacting the fertility, and therefore success, of honey bee queens. Queens collected from the field showed a clear negative relationship between viral counts and ovary mass—a trend which was observed in a second, independent set of queens. Furthermore, experimental infections resulted in a reduction in ovary mass and reduced vitellogenin expression, which suggests a causal, rather than merely correlative role of virus infection. Queens deemed to be failing by beekeepers have increased viral loads and smaller ovaries relative to queens perceived to be healthy. This finding combined with protein expression patterns indicating a decrease in oocyte development and increased antiviral response suggests that this is an economically relevant phenomenon.

Exactly what is responsible for this association between reduced ovary size and viral infections is yet unclear. It could be due to direct impacts of the viral pathology, or some indirect effect such as causing a queen to split resources between reproduction and launching an immune defense. The facts that each virus individually showed a similar effect size on ovary mass from the first survey queens, that an additive total viral count model better predicted ovary mass than each virus separately, and that experimental infections done with a different virus from those which we identified in the field provide evidence that this upregulation of the antiviral response and reduction in reproductive activity are general responses to viral immune activation, and not a specific response to a particular virus. Additionally, ovary mass is a highly plastic parameter—for example, it tends to decline when queens are confined to small cages with only a few attendants during shipping, but it rebounds again after being placed in a queen bank colony for several days or when she reinitiates oviposition^41^. Therefore, the effects that we observe on ovary size may be temporary and could conceivably be reversed if, for instance, the virus infection was cleared.

The increase in expression of antiviral HSPs we observed in naturally infected queens was recapitulated in queens of different ages experimentally infected with a different virus. Since some of the same antiviral proteins are upregulated with both virus infection and heat-shock, these data also highlight a potential mechanism by which extreme temperatures associated with climate change could have immunomodulatory effects. Whether such an interaction would have positive or negative impacts on reproduction remains to be tested. It is possible that pathogen spillover events, as has been suggested to occur between honey bees and other species, could negatively impact insects not only through direct fitness costs of infection, but through indirect effects on the population via reduced reproductive output.

## Materials and methods

### Queen surveys

For all surveys, beekeepers considered the queens to have ‘failed’ if they either displayed spotty brood patterns, were drone layers, or if the adult population rapidly dwindled, including the presence of chalkbrood infections that the colony could not resolve on their own. Beekeepers considered the queens to be ‘healthy’ if they produced robust brood patterns, the colony did not have signs of appreciable brood infections, and the adult population was strong. Additionally, queens were collected during the summer and beekeepers determined that the colonies were not in the process of gearing up to swarm.

For Survey 1, we utilized previously published data^35^, which included N = 105 queens (excluding imported queens, which were biased to smaller ovary sizes due to a longer caging duration). N = 93 of these queens had associated viral data. For the ovary mass comparison across surveys, we retained only queens from producers that met the following criteria: the producer collected both failed and healthy queens, queens were delivered to the laboratory in one shipment, and the producer provided n > 2 failed and healthy queens each. These criteria were applied because in the 2019 survey, producers from many different locations shipped queens to the laboratory by different methods and with collection schedules; therefore, queens spent different lengths of time in transit and in cages prior to transit, which we know from previous work impacts ovary mass. Applying these criteria reduced our sample size for the ovary mass comparison but removed these extraneous effects.

For Survey 2, N = 22 queens were collected from colonies in 2018 from three different apiaries in southeastern Pennsylvania. Healthy and failed queens were age- and apiary-matched in order to eliminate sampling bias. The queens were frozen on dry ice immediately in the field and shipped frozen to the University of British Columbia, at which time the queens were thawed on ice and dissected.

For Survey 3, queens were collected from local collaborating beekeepers in British Columbia as they became available during the summer of 2020. Apiary location, colony symptoms (if failed), genetic background (i.e., local or imported stock), and age were recorded. Failed and healthy queens were caged with candy and attendants then kept in a queen bank with plentiful nurse bees if they could not be immediately analyzed in the laboratory. The queens were transported to the University of British Columbia, where they were anesthetized with carbon dioxide, decapitated, and their spermathecae were removed for sperm viability analysis. The remaining queen bodies were stored at − 70 °C until further dissection to determine ovary mass.

### Sperm viability, sperm counts, and ovary mass

Sperm viability measurements for Survey 3 queens were obtained essentially as previously described^35^, following the methods of Collins and Donaghue^62^. Briefly, we burst the spermatheca with tweezers in 100 µl of Buffer D (17 mM d-glucose, 54 mM KCl, 25 mM NaHCO_3_, 83 mM Na_3_C_6_H_5_O_7_) and gently agitated the solution until sperm cells were homogeneously dispersed. We then stained the sperm using SYBR-14/propidium iodide dual fluorescent staining following the manufacturer’s protocol (Live/Dead sperm viability kit, Thermo). We dispensed 5 µl of the stained sperm into a well of a 24-well plate, over which we placed a round glass cover slip. We imaged the sperm (three fields of view per queen, average of ~ 100 sperm cells per image) using a Cellomics ArrayScan XTI (Thermo) and automatically counted live (green) and dead (red) channels using ImageJ v1.52a. Relative sperm abundances were obtained by counting the cumulative number of sperm across three fields of view. Every sample was analyzed in the same style of plate and with the same sized cover slip, so measuring the total sperm in a given area is proportional to total sperm in the sample. We later dissected the ovaries from the queen remains and weighed them using an analytical balance. Data for Survey 1 is already published and we direct the reader to McAfee et al.^35^ for the methods regarding the collection and processing of those queens.

### Viral quantification for queen surveys

Viral data (SBV, DWV-A, and BQCV) for Survey 1 were obtained by RT-qPCR exactly as previously described and are previously published data^35^. Briefly, samples were submitted to the National Bee Diagnostics Center, where total RNA was extracted from the head using a guanidinium isothiocyanate extraction buffer, cDNA was synthesized using the iScript cDNA synthesis kit (Bio-Rad Laboratories, Hercules, USA), and virus RNA was quantified by qPCR with previously published primers^35^. Standard curves for each virus amplicon were generated using serially diluted plasmids containing the target sequence. RP49 was used as the honey bee reference gene, enabling the viral RNA copy number per 30 ng of total RNA to be calculated. Copy numbers per 30 ng of RNA of all three viruses were added together to generate the ‘Total counts’ variable. All viral count data was then rescaled as counts per µg of extracted RNA in order to have data from both surveys depicted on the same scale, and log10 transformed. Queens from Survey 2 were not analyzed for viral infections because the sample size (22 queens) is expected to be too small to yield sufficient statistical power linking viral abundance to ovary size.

Queens from Survey 3 were processed by the Honey Bee Queen and Disease Clinic at North Carolina State University using methods as previously described^63^ with minor adjustments. For this sample set, we analyzed thoraxes because we have observed that other components of the head can sometimes interfere with PCR. We also tested for a wider range of viruses, including DWV-A, DWV-B, ABPV, SBV, IAPV, LSV, BQCV, and CBPV. Thoraxes were pulverized by bead beating and total RNA was extracted via the Zymo Research DirectZol RNA miniprep kit and Trizol reagent. RNA was quantified and verified for quality by NanoDrop Spectrophotometry and diluted to 200 ng/µL in RNAse/DNAse free water. cDNA was synthesized using the BioBasic All-In-One RT MasterMix. qPCR was performed on a QuantStudio 5 thermocycler using PowerUp Sybr Green chemistry. Standard curves were generated using serial dilutions of known quantities of custom plasmids containing the target sequences. Actin, 28 s ribosomal subunit, and GapDH were used as reference genes. Final copy numbers were normalized to reference gene values using GeNorm software^64^ and calculated per 3.33 ng of total RNA, then rescaled to counts per µg total RNA and log10 transformed.

### Statistical analyses of phenotypic data

All statistical analyses were performed in R v3.5.1. For Survey 3 data, we first checked if ovary mass, sperm count, and sperm viability were normally distributed and had equal variance between failed and healthy queens using the Shapiro and Levene test, respectively. The ovary mass and sperm viability passed these tests (*p* > 0.05); therefore, a linear mixed model (lme) was used for both, including health status (failed vs. healthy) and age (0, 1, or 2 years) as fixed effects and location (9 levels) as a random effect. The sperm viability data, however, was not normally distributed, so we analyzed these data with a generalized linear mixed model with a logit link function (glmer, family = binomial) as previously described^34^, constructed using the same fixed and random factors, but where sperm viability was represented as counts of ‘successes’ (live) and ‘failures’ (dead). Pooled ovary mass data from the three independent queen surveys were analyzed using a linear mixed model (lme) with health status as a fixed effect and queen source location as a random effect.

To determine the relationship between ovary mass and virus infection, we used a linear mixed effect model (R package lme4) fit by restricted maximum likelihood (REML) with queen source (apiary) as a random effect and viral counts (either individually, or as summed total viral load) and health status (healthy vs. failing) as fixed effects. We used the ‘DHARMa’ package for a simulation-based approach to assess the goodness of fit of these models by evaluating the scaled (quantile) residuals and tested the model for typical misspecification problems, such as over/underdispersion and residual spatial and temporal autocorrelation. To compare the individual virus vs. total viral load models we refit them using maximum likelihood (ML) in order to compare Akaike Information Criterion scores. Including source (breeder) as a random effect in the models for Survey 1 produced a singular fit with the breeder variance estimated as zero, but we chose to retain this part of the model because it is still a relevant, structural variable in the data and the differences between ovaries masses from each breeder are nontrivial.

### Ovary proteomic sample preparation and statistical analysis

Ovary samples from Survey 1 (N = 88, 5 samples from the original 93 queens were lost during processing) were prepared for mass spectrometry essentially as previously described^42^. One ovary from each queen was homogenized in a separate 2-ml screw cap tube containing 300 µl of lysis buffer (6 M guanidinium chloride, 100 mM Tris, pH 8.5) and four ceramic beads. The homogenizer (Precellys 24, Bertin Instruments) was set to 6500 s^−1^ for 30 s: samples were homogenized three times and placed on ice for 1 min between each. Then samples were centrifuged (16,000 relative centrifugal force, 10 min, 4 °C) to remove debris. 100 µL of supernatant was transferred to a new tube and diluted 1:1 with distilled H_2_O. Protein was precipitated by adding four volumes of ice-cold acetone and incubating overnight at − 20 °C. The precipitated protein was pelleted by centrifugation (6,000 relative centrifugal force, 15 min, 4 °C) and the supernatant was discarded. The protein pellet was washed twice with 500 µl of 80% acetone, then the pellet was allowed to air dry (~ 5 min) before solubilization in 100 µl of digestion buffer (6 M urea, 2 M thiourea). Approximately 20 µg of protein was reduced (0.4 µg dithiothreitol, 20 min), alkylated (2 µg iodoacetamide, 30 min, dark), and digested (0.4 µg Lys-C for 2.5 h, then 0.5 µg trypsin overnight). Digested peptides were acidified with one volume of 1% trifluoroacetic acid and desalted with high-capacity STAGE tips as previously described^65^. Eluted samples were dried (SpeedVac, Eppendorf, 45 min) and resuspended in Buffer A (0.1% formic acid, 2% acetonitrile), then peptide concentrations were determined using a NanoDrop (Thermo, 280 nm). LC–MS/MS data acquisition was performed as previously described^42^.

MS data was searched using MaxQuant (v1.6.8.0) with the parameters and database as previously described^42^. In Perseus (v 1.5.5.3), the resulting protein groups were filtered to remove reverse hits, contaminants, proteins identified only by site, and proteins identified in fewer than 10 samples. Normalized LFQ intensity was then log2 transformed. Subsequent analyses were performed in R (v3.6.3). Differential expression analysis was performed using the limma package in R^66^ with ovary mass, status (failed vs. healthy), and total viral counts (log10 transformed of combined RT-qPCR counts, + 1 to avoid undefined terms) included as fixed effects. Empirical Bayes moderation of the standard errors was then performed on the resulting linear model, and finally the false discovery rate was controlled to 5% using the Benjamini–Hochberg correction. GO term enrichment analysis was performed on the proteins differentially expressed among status groups using Ermine J as previously described^41^, though no terms were significant. We used a linear model with ovary mass, status, and total viral counts as fixed effects to analyze the expression of ApoLp-I/II and ApoLp-III.

### Statistical analysis of spermathecal proteomic data

We analyzed previously published proteomics data^35^ using the limma R package essentially as previously described for ovaries. Proteomics data were first groomed to remove samples that did not have associated viral abundance data. We included log10 transformed total viral load (+ 1 to avoid undefined terms), health status (failed or healthy), and sperm counts as fixed effects in the statistical model. We used a Benjamini–Hochberg correction to control the false discovery rate 5%. We direct the reader to McAfee et al*.*^35^ for details on proteomics sample preparation and upstream data processing.

### IAPV experimental infections for measuring ovary mass

Mated queens of approximately the same age (< 1 year old, roughly ~ 1–2 months) were obtained in a single shipment from California. Queens were anesthetized with CO_2_ for 6 min before being injected between the abdominal tergites with approximately 4nL of IAPV-inoculum containing ~ 1400 copies of IAPV (determined by absolute quantification using qPCR) (n = 10) or phosphate buffer saline (PBS) as mock (n = 10). Injections were performed using the FemoJet Microinjector (Eppendorf) with hand-pulled glass needles. The queens were then kept in wooden cages with 5 attendant workers and provided with sugar candy in the incubator at 34 °C with a water pan for humidity. After 65 h the queens were sacrificed, and the wet ovary masses measured on an analytical balance. IAPV levels were quantified in the heads of all queens by the National Bee Diagnostic Center using the same methods as described above for the viral analysis of the queen surveys. The mock-injected queens were additionally analyzed for DWV, SBV, and BQCV.

### IAPV experimental infections for expression analysis

Three large groups of sister queens were produced from a healthy mother queen to study the direct effect of virus infection on queens of three different ages following honey bee queen-rearing technique^67^. Briefly, young larvae (12–24 h old) were grafted into artificial queen cups and placed in a populous but queenless nurse colony to develop. Prior to grafting, the donor colony was visually determined to be free of symptomatic diseases (Nosemosis, Varroosis, European foulbrood, American foulbrood, and Chalkbrood). After 6 days, sealed queen cells were transferred to an incubator (35 °C, 65% humidity) and treated as follow:

The first group was used for pupal infections. Two days before emergence, queen pupae were gently removed from their capped cells. These pupae were either injected with 2 µL of IAPV-inoculum containing 5.24 × 10^3^ virus particles (n = 34), or phosphate buffered saline (PBS) as a mock control (n = 31). Injections were performed using a Nanojet™ syringe pump (Chemix, USA) with an infusion flow rate of 0.1 µL/s, where the needle was inserted into the first abdominal segment located immediately behind the metathorax. Each queen then was placed individually in a 15 mm well in the 24 well glass bottom cell culture plate and kept in the incubator (35 °C, 65% humidity). While in the incubator, development of the virus injected queens was compared with the PBS injected queens. Our observations confirmed a complete cessation of development in IAPV injected queens 20 h post injection, which was not observed in the control group. Therefore, the experiment was terminated, and the abdomen of each queen was then separated, placed in an Eppendorf tube, and stored in a freezer at − 80 °C until RNA extraction.

The second group of queens was used for infecting newly emerged queen in the pre-reproductive stage. Two days after emergence, queens were either injected with the IAPV-inoculum (n = 30), or PBS as mock control (n = 30) as explained above. Each queen was accompanied with 10–15 worker honey bees from the donor colony in a single-use plastic cup, provided with water and sugar candy. Cups of IAPV-inoculated and PBS-injected groups were maintained separately in the incubator (35 °C, 65% humidity). The IAPV-inoculated queens started showing paralysis symptoms around 20–24 h post injection. The experiment was terminated at the onset of symptoms, and after anesthetization using CO_2_ the queens’ abdomens were sampled and stored as described above.

For infection of the last group of reproductive queens, right after emergence, queens were caged and banked in a donor colony for one week. Then, they were taken out and treated with CO_2_ two times a week later and return to the donor colony for one more week to stimulate ovary development without mating^48^. Thereafter, queens were either injected with the IAPV-inoculum (n = 34), or PBS as mock control (n = 31) and maintained with attendant workers in the incubator (35 °C, 65% humidity), as described above. Paralysis symptoms started in the IAPV-inoculated queens 40–44 h post injection, when the experiment was terminated. Abdomens were sampled as described above.

### RNA isolation, cDNA Synthesis, and Quantitative PCR (qPCR) on experimentally infected queens

Each abdomen was transferred into a 2 mL Ruptor homogenizing tube containing 5 ceramic beads. The samples were then homogenized using the automated Bead Ruptor Elite (Omni International, Kennesaw, GA, USA) at a speed of 5 m per second (5 m/s) for 20 s. Followed by homogenization, total RNA was extracted using an established TRIzol™ (Invitrogen, Carlsbad, CA, USA) protocol. The concentration and purity of the extracted RNA samples were measured using a Nanodrop OneC Microvolume UV–Vis Spectrophotometer (Thermo Fisher Scientific, MA, USA). The total RNA concentration was adjusted to 20 ng/µL in molecular grade water (Fisher Scientific, Fair Lawn, NJ, USA). Then, a two-step quantitative qPCR assay was carried out to quantify the target genes. For each sample, cDNA was synthesized using the High-Capacity cDNA Reverse-Transcription Kit (Applied Biosystems, Foster City, CA, USA). Ten microliters of the RNA template (total, 200 ng) were added to 10 µL of the provided cDNA master mix, followed by an incubation period as recommended by the manufacturer; 10 min at 25 °C, 120 min at 37 °C, and 5 min at 85 °C. The resulting cDNA solution then diluted tenfold in molecular grade water to serve as template in subsequent qPCR. The qPCR was conducted in duplicate using 384-well plates on a QuantStudio™ 6 cycler (Thermo Fisher Scientific). The reactions were performed using unlabeled primers and SYBR Green DNA binding dye (Applied Biosystems) with a volume of 12 µL with the final primer concentrations of 0.4 µM. We added RNase-free water as template for a No Target Control (NTC) and a No Reverse Transcriptase (NRT) control as an additional negative control^68^. The thermal cycling conditions were 10 min at 95 °C, followed by 40 cycles consisting of a denaturing stage at 95 °C for 15 s and an annealing/extension stage at 60 °C for 1 min. This procedure was followed by a final melt-curve dissociation analysis to confirm the specificity of the products. For IAPV, the C_q_ values were determined at the same fluorescence threshold (0.05) for all plates, and a C_q_ value of 35 or lower was recorded as positive amplification. For three genes of interest and three housekeeping genes we collected C_q_ values based on the default threshold as determined by the QuantStudio™ 6 for each target gene. Fluoresence measurements were taken at the end of each cycle.

We quantified the IAPV intensity and the expression level of three genes of interest including vitellogenin, protein lethal(2)essential for life-like, heat shock protein 70 cognate 4 (see Supplementary Table S5 for primer sequences). Three housekeeping genes including actin, glyceraldehyde 3-phosphate dehydrogenase (GapDH) and ribosomal protein S5 (RPS5) were also amplified as internal control and for relative quantification. The primers used in this experiment have previously been validated to detect the intended targets and are commonly used in honey bees^69,70^. We quantified immune-gene expression for each sample using relative quantification^71^. We calculated geometric mean of the C_q_ value from three reference genes (RPS5, actin, and GapDH) to confirm amplification. We subtracted the C_q_ value of geometric mean from target gene for each sample (∆C_q_ = C_q_(gene of interest) − C_q_(GMean of reference genes)). To calculate the ∆∆C_q_ for each target gene, we subtracted the average of the ∆C_q_ values across the control samples at each age from the ∆C_q_ for each target gene. The fold-change in gene expression was then calculated using the 2^−(∆∆Cq)^ method. Pairwise comparisons of gene expression were evaluated using the wilcox.test function in the ggpubr package (v0.4.0). Data are plotted in the form of log_10_(fold change in gene expression) for clarity.

## Supplementary Information

Supplementary Information 1.

Supplementary Information 2.

## Data Availability

All data underlying the figures in this manuscript are supplied as supplementary information. The previously published (spermathecal fluid) raw data are publicly available on the MassIVE archive (www.massive.ucsd.edu, accession MSV000085428). The ovary mass spectrometry data are available on the MassIVE archive (www.massive.ucsd.edu, accession MSV000087150). Source code underlying figures and data analysis are available freely upon request.
