# Supplementary material for: Fertility costs of cryptic viral infections in a model social insect
Source: Sci Rep. 2022 Sep 23;12:15857. doi: 10.1038/s41598-022-20330-4 (PMC9508145; doi:10.1038/s41598-022-20330-4)
Supplement: Supplementary file 1 — Supplementary Information 1. [file 41598_2022_20330_MOESM1_ESM.pdf]

Abigail Chapman[1*], Esmaeil Amiri[2], Bin Han[3], Erin McDermott[4], Olav Rueppell[5], David R Tarpy[4], Leonard J Foster[1*], and Alison McAfee[1]

1. Department of Biochemistry and Molecular Biology, Michael Smith Laboratories, University of British Columbia, Vancouver, British Columbia, Canada
2. Delta Research and Extension Centre, Mississippi State University, Stoneville, MS, USA
3. Institute of Apicultural Research, Chinese Academy of Agricultural Sciences, Beijing, China
4. Department of Entomology and Plant Pathology, North Carolina State University, Raleigh, North Carolina, USA
5. Department of Biological Sciences, University of Alberta, Edmonton, Alberta, Canada

*Correspondence: abbi.a.chapman@gmail.com, foster@msl.ub.ca

Supplementary Results

Confirmation of IAPV infection in experimental innoculations

We infected queen pupae, two day old adult queens and two week old adult queens via injection as described in the Methods, and confirmed the presence of infection using RT-qPCR (**Figure S1**).

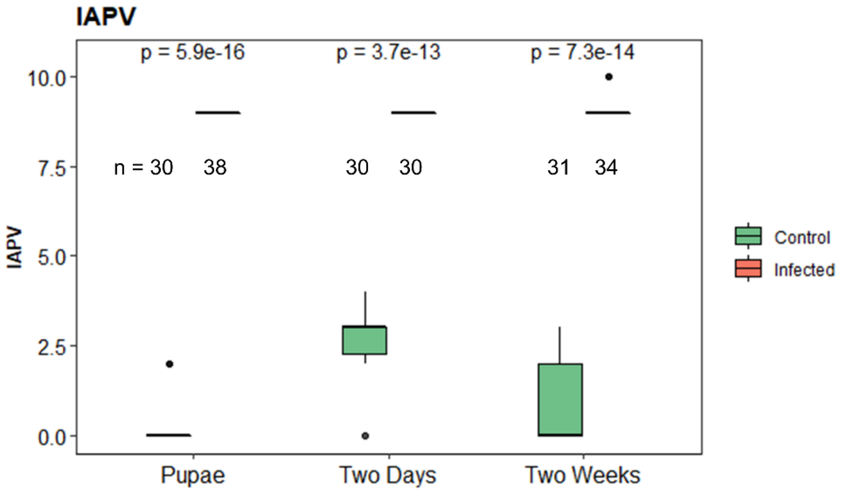

Figure S1. Confirmation of IAPV infection in experimentally infected queens. IAPV load in each sample was quantified using absolute quantification, based on our standard curves obtained through serial dilutions of known numbers of amplicons as described before [1]. To improve data compliance with parametric assumptions, statistical tests were performed on transformed raw data, according to $x' = log10(x+1)$. The y axis indicates log10 transformed viral RNA copies.

References

1. Francis RM, Nielsen SL, Kryger P: **Varroa-virus interaction in collapsing honey bee colonies**. *PLoS One* 2013, **8**(3):e57540.
